# The thorax of the cave cricket *Troglophilus neglectus*: anatomical adaptations in an ancient wingless insect lineage (Orthoptera: Rhaphidophoridae)

**DOI:** 10.1186/s12862-016-0612-5

**Published:** 2016-02-18

**Authors:** Fanny Leubner, Thomas Hörnschemeyer, Sven Bradler

**Affiliations:** Department of Morphology, Systematics & Evolutionary Biology, J-F-Blumenbach Institute for Zoology & Anthropology, Georg-August-University Göttingen, Göttingen, Germany

**Keywords:** Orthoptera, Ensifera, Rhaphidophoridae, Winglessness, Morphology, Phylogeny

## Abstract

**Background:**

Secondary winglessness is a common phenomenon found among neopteran insects. With an estimated age of at least 140 million years, the cave crickets (Rhaphidophoridae) form the oldest exclusively wingless lineage within the long-horned grasshoppers (Ensifera). With respect to their morphology, cave crickets are generally considered to represent a `primitive’ group of Ensifera, for which no apomorphic character has been reported so far.

**Results:**

We present the first detailed investigation and description of the thoracic skeletal and muscular anatomy of the East Mediterranean cave cricket *Troglophilus neglectus* (Ensifera: Rhaphidophoridae). *T. neglectus* possesses sternopleural muscles that are not yet reported from other neopteran insects. Cave crickets in general exhibit some unique features with respect to their thoracic skeletal anatomy: an externally reduced prospinasternum, a narrow median sclerite situated between the meso- and metathorax, a star-shaped prospina, and a triramous metafurca. The thoracic muscle equipment of *T. neglectus* compared to that of the bush cricket *Conocephalus maculatus* (Ensifera: Tettigoniidae) and the house cricket *Acheta domesticus* (Ensifera: Gryllidae) reveals a number of potentially synapomorphic characters between these lineages.

**Conclusions:**

Based on the observed morphology we favor a closer relationship of Rhaphidophoridae to Tettigoniidae rather than to Gryllidae. In addition, the comparison of the thoracic morphology of *T. neglectus* to that of other wingless Polyneoptera allows reliable conclusions about anatomical adaptations correlated with secondary winglessness. The anatomy in apterous Ensifera, viz. the reduction of discrete direct and indirect flight muscles as well as the strengthening of specific leg muscles, largely resembles the condition found in wingless stick insects (Euphasmatodea), but is strikingly different from that of other related wingless insects, e.g. heel walkers (Mantophasmatodea), ice crawlers (Grylloblattodea), and certain grasshoppers (Caelifera). The composition of direct flight muscles largely follows similar patterns in winged respectively wingless species within major polyneopteran lineages, but it is highly heterogeneous between those lineages.

**Electronic supplementary material:**

The online version of this article (doi:10.1186/s12862-016-0612-5) contains supplementary material, which is available to authorized users.

## Background

The evolution of wings is considered to be a key innovation responsible for the unrivaled evolutionary success of insects, improving dispersal capability, predator avoidance, as well as the access to scattered food sources and mating partners [1]. Beyond flight, wings can provide additional advantages, contributing to thermoregulation, defensive behavior and acoustic communication [2–4]. Yet, wing loss is a common phenomenon among pterygotes [1]. In Ensifera (long-horned grasshoppers), one of the most species-rich lineages among the Polyneoptera, wings are often reduced to tiny remnants whose only purpose appears to be the production of sound [5, 6]. Orthoptera in general have long been of interest to scientists studying intra-specific acoustic communication and hearing systems. Crickets (Gryllidae) and bush-crickets or katydids (Tettigoniidae) in particular are well known for their elaborate acoustic signaling via tegminal stridulation that is associated with mating and territorial behavior [4]. In the last century, numerous biologists dedicated their research to bioacoustics and countless studies have been conducted illuminating the neuroanatomical [7, 8], behavioral [9] and evolutionary [10, 11] background of ensiferan bioacoustics.

Some ensiferan taxa have completely reduced their wings, nevertheless. To understand the evolution of bioacoustics within the Ensifera special attention was paid to these wingless and deaf taxa, such as the Rhaphidophoridae, commonly known as camel and cave crickets. The neuroanatomy of their chordotonal organs [10] as well as their vibratory communication through low frequencies [12] is assumed to reflect the ancestral condition of bioacoustics within the Ensifera. Also in regard of their overall morphology, cave crickets are considered a ´primitive` lineage among Ensifera preserving several characters in their plesiomorphic state, e.g. the morphology of the ovipositor, the absence of tarsal pulvilli and the absence of posterofurcal connectives in the thorax [13]. With about 550 described species, these insects form an ecologically specialized group mainly adapted to cave life [5]. Rhaphidophoridae has a disjunct geographical distribution restricted to the temperate areas of the Northern and Southern hemispheres as reflected by their phylogeny [14]. Rhaphidophoridae comprises two major groups: Rhaphidophorinae, distributed in Eurasia and North America, and Macropthinae that is restricted to South Africa, South America and New Zealand [15, 16]. Although the monophyly of Rhaphidophoridae is well supported in molecular analyses [17–20], cladistic analyses of morphological characters indeed could not identify any supporting apomorphy for this clade yet [21, 22]. The species *Troglophilus neglectus* investigated in this study appears to branch off from a basal node, forming the sister taxon to the remaining Rhaphidophoridae [19]. In this respect, *T. neglectus* likely retains characters from the last common ancestor of Rhaphidophoridae and can be considered representative for this taxon in general.

Numerous hennigian (mental) and cladistic studies of Ensifera including Rhaphidophoridae have led to competing hypotheses with respect to the relative positions of the two most species-rich groups within the Ensifera, the true crickets (Gryllidae) and the bush-crickets (Tettigoniidae) (Additional file 1). Traditionally, ensiferan taxonomy is based on the morphology of wings and wing venation in particular. Interestingly, the phylogenetic hypotheses based on this specific character complex differ remarkably. Following the classification scheme of Handlirsch [23], Zeuner [24] proposed a closer relationship of crickets (‘Grylloidea’ therein) and bush-crickets (‘Tettigoniidae’ therein) and considered both taxa as having evolved from different fossil representatives of the Prophalangopsidae. He considered the tegminal stridulation and its specific wing morphology as an apomorphic character in the last common ancestor of crickets and bush-crickets. On the other hand, Karny [25, 26] and Sharov [27] shared the opinion that the true crickets and relatives (mole crickets, Gryllotalpidae, and antloving crickets, Myrmecophilinae) originated from the gryllacridids (including Rhaphidophoridae), whereas the bush-crickets (Tettigoniidae) were assumed to form an independent lineage within the Ensifera. However, the majority of hennigian and cladistic morphological studies [13, 21, 22, 28] as well as phylogenetic analyses based on molecular data [19, 29–33] propose a division of the Ensifera in two major groups: the “grylloid” clade, including true crickets (Gryllidae), mole crickets (Gryllotalpidae) and antloving crickets (Myrmecophilinae), and a “tettigonioid” clade, comprising the bush-crickets (Tettigoniidae), cave crickets (Rhaphidophoridae), wetas (Anostostomatidae), Jerusalem crickets (Stenopelmatidae) and raspy crickets (Gryllacrididae). Dune crickets (Schizodactylidae) are assigned to either of these two clades according to different authors [21, 22].

While studies solely based on molecular data may provide a robust phylogenetic framework for any given organismic group, comparative morphological research is essential for interpreting evolutionary scenarios [34] and tracing functional transformations and adaptations [35]. In particular, the morphology of insect thoraces has repeatedly played a substantial role in understanding the systematics and evolution of certain insect groups [36–39]. In Ensifera this character complex is hitherto insufficiently studied, with publications that either give only a scarce description of the thoracic skeleton and/or merely include a part of the thoracic musculature. Very few detailed investigations of ensiferan thoraces provide characterizations of skeletal structures in addition to a complete description of the muscular equipment. These studies only consider representatives of the most species-rich ensiferan lineages: Voss [40–43] gives an exceedingly detailed description of the thorax of the house cricket *Acheta domesticus* (Gryllidae), whereas Maki [44] provides the only existing description of the thoracic musculature of a bush-cricket, *Conocephalus maculatus* (Tettigoniidae). Studies focusing on the thoracic morphology of Rhaphidophoridae are scarce. Carpentier [45] gives a brief description of the thoracic skeleton of the greenhouse stone cricket *Diestrammena asynamora* (Rhaphidophorinae) in addition to a study of its pleural musculature [46]. Furthermore, Richards [47] presents a fragmentary description of the thoracic morphology of *Macropathus filifer*, a rhaphidophoridean species belonging to the southern group Macropathinae.

Here we present a detailed description of the skeletal structures and the muscular equipment of the thorax of the East Mediterranean cave cricket *Troglophilus neglectus* (Rhaphidophorinae). The thoracic morphology of *T. neglectus* is compared to the conditions found in other representatives of Orthoptera in order to detect possible apomorphic traits of Rhaphidophoridae. Furthermore, the investigated character complex is evaluated in the context of its phylogenetic information content, and potential synapomorphies of the competing phylogenetic hypotheses of ensiferan relationships are discussed. Moreover, the general nomenclature recently proposed for thoracic musculature of Neoptera [36] is critically reviewed in light of our results. It is evident that within the Neoptera wings were lost several times independently in evolution and this was a step-like process with numerous morphological transformations in each lineage. Therefore, our observations are compared to the thoracic morphology of other wingless polyneopteran representatives, such as Zoraptera [36], Mantophasmatodea [48] or Phasmatodea [49] in order to compile common adaptations of the thoracic skeletal and muscular system related to secondary winglessness. Based on our novel anatomical data we will provide a detailed description of the consequences of wing loss on the functional anatomy of insect thoraces and thoroughly address the question whether these transformations follow a similar pattern.

## Methods

### Specimens

The specimens investigated in this study were collected in Brje pri Komnu, Slovenia, in July 2008 and identified as *Troglophilus* (*Paratroglophilus*) *neglectus* Krauss, 1879 [50]. All specimens were preserved in 70% ethanol. For the sake of consistency in subsequent comparative studies, all investigated specimens are female adults. In total, four individuals were investigated using the following different methods.

### High-resolution photography

Three specimens were used to investigate and illustrate the thoracic skeleton. One complete and undamaged specimen was dehydrated in a graded ethanol series and critical-point dried (Balzer CPD 030) to visualize the outer lateral and dorsal view. Another specimen was sagitally cut and macerated in 5% KOH (1 h in a heating cabinet with 60 °C) and likewise dried at critical point. Critical-point drying was applied to improve the contrast of the thoracic sclerites against the membranous areas and to visualize the sclerites in more detail. One specimen was fixed in a ventrally overstretched position to expose the neck region and subsequently dried using the HMDS (Hexamethyldisilazane, Carl Roth GmbH & Co KG, item number 3840.2) procedure [35]. Photographs of the HMDS-dried specimen were taken using a digital camera (OLYMPUS Pen E-P2) mounted on a stereomicroscope ZEISS Stemi SV11. The critical-point dried specimens were photographed with a CANON EOS 550D equipped with a macro lens (100 mm) and a ring flash (METZ 15 MS-1). The overall sharp images are composed of image stacks edited in Helicon Focus® (Helicon Soft) and Adobe Photoshop® CS3.

### Synchrotron radiation micro computer tomography (SRμCT) and 3D-reconstruction

In order to investigate the thoracic musculature, one specimen was dehydrated in a graded ethanol series, critical-point dried (Balzer CPD 030) and mounted on a specimen holder (aluminium stub). The scan was performed at the synchrotron radiation facility BESSY II (Berlin, Germany). The three-dimensional model of the thorax was created using AMIRA®5.4.3 and Autodesk Maya® 2013. Rendered images were edited using Adobe Illustrator® CS3.

### Terminology

The terminology of the thoracic skeleton largely follows Snodgrass [51] and Friedrich & Beutel [36]. Terms used by authors of ensiferan-specific literature e.g. [13, 40] are mentioned in the case of inconsistency. The thoracic musculature of *Troglophilus* (*Paratroglophilus*) *neglectus* is described and muscles are numbered consecutively. We homologize the observed muscles in *Troglophilus*, in addition to that of two other ensiferans, *Conocephalus maculatus* [44] (*Xiphidion maculatum* therein) and *Acheta domesticus* [41] (*Gryllus domesticus* therein) with the muscles described following the nomenclature of Friedrich & Beutel [36] for neopteran insects, allowing for comparison to studies of other authors. The distinctive set of thoracic muscles found in *Troglophilus* is compared with the condition in other polyneopteran taxa, i.e. two grasshoppers (Caelifera), *Locusta migratoria migratorioides* [44] (*Locusta migratoria manilensis* therein) and *Atractomorpha sinensis* [44] (*Atractomorpha ambigua* therein), two stick insects (Phasmatodea), *Carausius morosus* [52] (*Dixippus morosus* therein) and *Megacrania tsudai* [53], and one heelwalker (Mantophasmatodea), *Austrophasma caledonensis* [48]. The current taxonomy of the examined species follows Eades et al. [54] and Brock [55].

## Results

### Skeleton

**Fig. 1:**
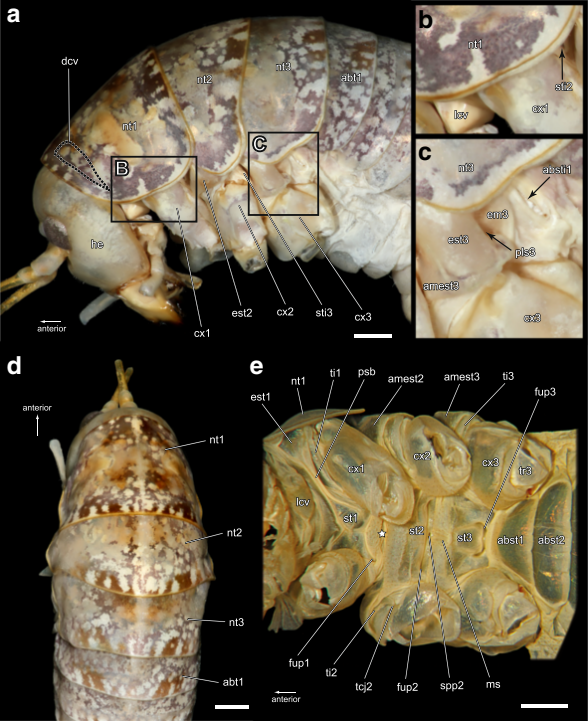
Exterior view of the thoracic skeleton of *Troglophilus neglectus*, legs removed. **a** Lateral view of left body side. The position of the dorsal cervical sclerite (dcv) is marked by the dashed line. (**b**), (**c**) Enlarged details of the cervical and thoracic pleural region as indicated in (**a**). **d** Dorsal view. **e** Ventral view. The white asterisk marks the invagination point of the prospina. The specimen figured in (**a**)–(**d**) is critical-point dried; the specimen depicted in (**e**) is dried with HMDS in an overstretched position to provide visibility of the cervical region. abst1/2, first/second abdominal sternum; absti1, first abdominal stigma; abt1, first abdominal tergum; amest2/3, anterior margin of mes-/metepisternum; cx1/2/3, pro-/meso-/metacoxa; dcv, dorsal cervical sclerite; em3, metepimeron; est1/2/3, pro-/meso-/metepisternum; fup1/2/3, furcal pit of pro-/meso-/metasternum; lcv, lateral cervical sclerite; ms, median sclerite; nt1/2/3, pro-/meso-/metanotum; pls3, metathoracic pleural suture; psb, pleuro-sternal bridge; spp2, mesospinal pit; st1/2/3, pro-/meso-/metasternum; sti2/3, meso-/metathoracal stigma; tcj2, trochantino-coxal joint of mesothorax; ti1/2/3, pro-/meso-/metatrochantin; tr3, metatrochanter. Scale bars: 1 mm

The thorax of *T. neglectus* comprises approximately two thirds of the total body length and is strongly curved downwards with the dorsal side nearly two times longer than the ventral side. The sclerites are colored light brown, speckled with dark reddish brown. All thoracic terga are ventrally elongated and saddle-shaped, masking great parts of the thoracic pleura in a lateral view (Fig. 1a). Wings and wing base sclerites are lacking. The phragmata are weakly developed and function as attachment points for the poorly developed dorsal longitudinal muscles. Ventrally, the anterior parts of the sterna, the membranous areas between these sclerites, and the inner surfaces of the coxae are covered by numerous setae (Fig. 1e).

### Prothorax

**Fig. 2:**
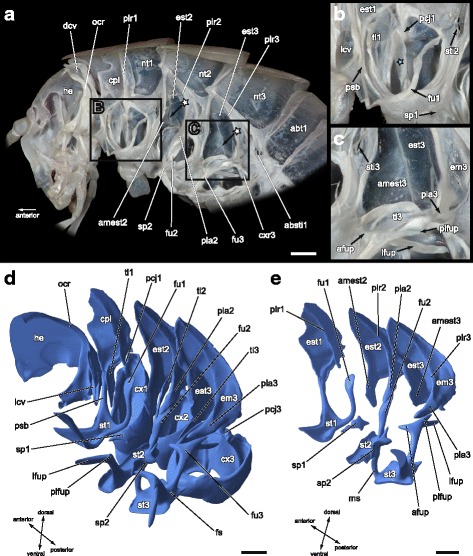
Interior view of the thoracic skeleton of *T. neglectus*. (**a**)–(**c**) Photographs, (**d**)–(**e**) Three-dimensional reconstruction of skeletal elements of right half of thorax based on SRμCT-sections. **a** Lateral view of right body half. White asterisks mark the strongly sclerotized edge between episternum **est** and its anterior margin **amest. b** Detail of prothoracic sternopleural region. The blue asterisk marks the tendon of muscle 11 (Idvm19). **c** Detail of metathoracic sternopleural region. **d** Inner posterolateral view, terga removed. **e** Inner posterolateral view, showing sternal and pleural skeletal elements, only. absti1, first abdominal stigma; abt1, first abdominal tergum; afup, anterior furcal process; amest2/3, anterior margin of mes-/metepisternum; cpl, cryptopleura; cx1/2/3, pro-/meso-/metacoxa; cxr3, metacoxal rim; dcv, dorsal cervical sclerite; em3, metepimeron; est1/2/3, pro-/mes-/metepisternum; fu1/2/3, pro-/meso-/metafurca; he, head; lcv, lateral cervical sclerite; lfup, lateral furcal process; ms, median sclerite; nt1/2/3, pro-/meso-/metanotum; ocr, occipital rim; pcj1/2/3, pleurocoxal joint of pro-/meso-/metathorax; pla2/3, meso-/metathoracic pleural arm; plfup, posterolateral furcal process; plr1/2/3, pro-/meso-/metathoracic pleural ridge; psb, pleurosternal bridge; sp1/2, pro-/mesospina; st1/2/3, pro-/meso-/metasternum; sti2/3, meso-/metathoracal stigma; ti1 /2/3, pro-/meso-/metatrochantin. Scale bars: 1 mm

**Fig. 3:**
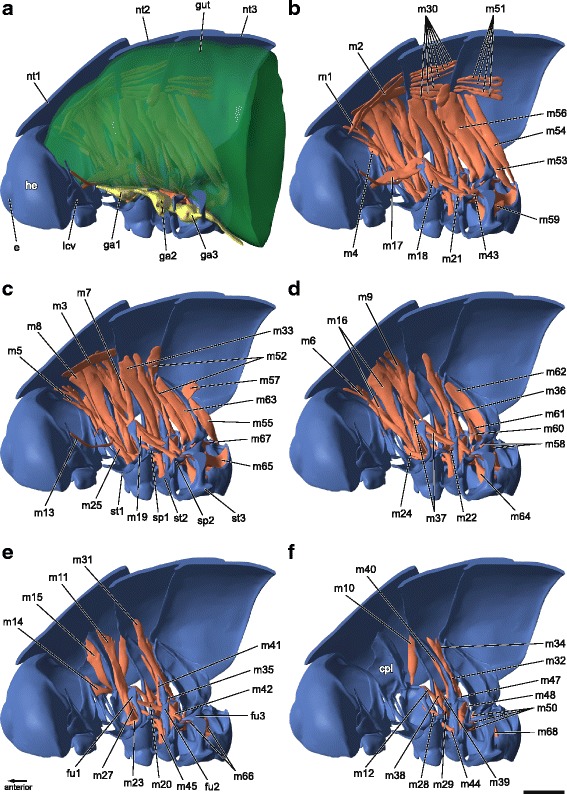
Thoracic skeletomuscular system of *T. neglectus*. Three-dimensional reconstruction of right half of thorax based on SRμCT-sections. Muscles: red; skeleton: blue; digestive tract: green; nervous system: yellow. Virtual dissection (**a**–**f**). cpl, cryptopleura; e, compound eye; he, head; lcv, lateral cervical sclerite; nt1/2/3, pro-/meso-/metanotum; fu1/2/3, pro-/meso-/metafurca; ga1/2/3, pro-/meso-/metathoracic ganglion; sp1/2, pro-/mesospina; st1/2/3, pro-/meso-/metasternum. For muscle terminology see text and Table 1. Scale bar: 1 mm

**Fig. 4:**
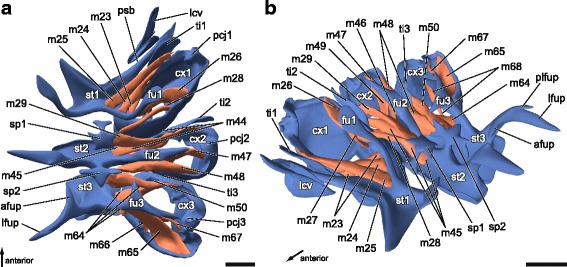
Sternocoxal muscles (scm) of *T. neglectus*. Three-dimensional reconstruction based on SRμCT-sections. **a** Dorsal view. **b** Anterolateral view. afup, anterior furcal process; cx1/2/3, pro-/meso-/ metacoxa; fu1/2/3, pro-/meso-/metafurca; lcv, lateral cervical sclerite; lfup, lateral furcal process; pcj1/2/3, pleurocoxal joint of pro-/meso-/metathorax; plfup, posterolateral furcal process; psb, pleurosternal bridge; sp1/2, pro-/mesospina; st1/2/3; pro-/meso-/metasternum; ti1/2/3, pro-/meso-/metathoracic trochantin. For muscle terminology see text and Table 1. Scale bars: 500 μm

An extensive cervical membrane connects the thorax to the head capsule. Several sclerites stabilize the cervical membrane and function as articulated connections between the head and the prothorax. The single lateral cervical sclerite **lcv** on each side consists of two connected parts being arcuate towards each other on the ventral side (Figs. 1a, b; 2b, d). The anterior part is of nearly triangular shape, the longest edge projecting medially. The anterior part extends dorsally to a slender, well sclerotized process, which articulates laterally with the occipital rim **ocr** of the head (Fig. 2d). The posterior part of the lateral cervical sclerite is triangular and its dorsal part articulates with the pleurosternal bridge **psb** of the prothorax (Fig. 2d). The unpaired dorsal cervical sclerite **dcv** is weakly sclerotized and situated in the upper half of the cervical membrane (Figs. 1a; 2a). This sclerite has a clip-like appearance reminiscent of a headband, widened at the dorsal side, narrowing strongly towards the ventral side. It is completely covered by the saddle-shaped pronotum **nt1** (Fig. 1a) and only visible when the neck membrane is overstretched. The pronotum has a smooth surface without distinct ridges or grooves. It is laterally extended and bent ventrally, covering most of the propleura. The posterior part of the pronotum overlaps the mesonotum **nt2** (Fig. 1a, d). At the ventral side, the pronotum is continuous with an inward directed membranous fold that is connected to the exterior face in the lower third of the cryptopleura **cpl** (*Pleurallamelle* in [40]). The cryptopleura is sail-shaped (Fig. 2a, d). The pleural suture divides the cryptopleura in an anterior episternum and a posterior epimeron. The inner propleural ridge **plr1** is well developed and forms the pleurocoxal articulation **pcj1** at its ventral tip with the lateral procoxal rim (Fig. 2). The proepisternum **est1** is distinctly larger than the narrow proepimeron, which is merely the posterior part of the pleural ridge. The upper part of the proepisternum is thin and broadened and serves as an attachment point for several pleurocoxal muscles (m14–m16; see Fig. 3d, e). The lower part of the proepisternum **est1** bears a vesicular protrusion (Fig. 2b), which is the only visible part of the cryptopleura from an outer ventrolateral view. The anterior ventral angle of the proepisternum is continuous through the pleurosternal bridge **psb** (*precoxal bridge* in [56]; *Coxosternum* in [40]) with the anterior lateral angle of the prosternum **st1** (Fig. 2). The prosternum is nearly rectangular, but it shows a constriction along the ventromedian axis (Figs. 1e; 2d). The prosternal margins appear as strongly sclerotized ridges. The lateral and posterior ridges converge at each posterolateral corner of the prosternum and bear the inner profurca **fu1** (Fig. 2b, d). The profurca consists of a slender stem, which extends to a laterally orientated, shovel-shaped profurcal arm. From the exterior no spinasternum is recognizable (Fig. 1e). However, the internally located prospina **sp1** is well developed. It has a star-like shape from a top view with paired anterolateral and posterolateral processes and an unpaired anterior process (Fig. 2e). The feather-shaped prothoracic trochantin **ti1** is exposed in front of the coxal rim. Its ventral tip articulates with the anteromedian part of the procoxa **cx1** (Fig. 2b, d). Two sternocoxal muscles (m27, m28) are attached to inner processes of the large oval procoxal rim, one mediad and one laterad (Fig. 4).

### Mesothorax

The meso- and metathorax are almost identical in size. Like the pronotum **nt1**, also the pterothoracic nota **nt2/nt3** show no external or internal sculpturing and are ventrally elongated covering the most part of the pterothoracic pleura (Fig. 1a, d). The mesopleura has a triangular form tapering at the dorsal side. The mesepisternum **est2** is much broader than the epimeron **em2** (Fig. 2). The mesepisternum is folded inwards at the anterior edge projecting into a median direction in an obtuse angle. This inwardly folded part of the episternum is referred to as anterior margin **amest2** (Fig. 2a, e) and serves as an attachment area for several muscles (m38, m39). The anterior edge of the mesepisternum, connecting the episternum with its anterior margin, is forming a strongly sclerotized ridge (marked by white asterisks in Fig. 2a). The anterior margin of the mesepisternum extends medially onto the level of the trochantinocoxal joint. A massive and long pleural arm **pla2** protrudes from the straight mesopleural ridge **plr2** (Fig. 2d, e). A sclerotized bridge between the pleura and the sternum is absent in the mesothorax. The mesosternum **st2** has a trapezoid shape, the longer edge orientated towards the head. The margins of the mesosternum are relatively indistinct because it is not delimited by strongly marked ridges as is the prosternum. The furcal pit **fup2** and the spinal pit **spp2** are located along a longitudinal groove at the posterior margin of the mesosternum **st2** (Fig. 1e). The mesothoracic furca **fu2** has a long lateral process **lfup** and a short posterolateral process **plfup** (Fig. 2d). The form of the mesothoracic spina **sp2** is reminiscent of a butterfly with expanded wings consisting of paired dorsolateral and ventrolateral processes and an unpaired posterodorsal one (Figs. 2d, e; 4b). The mesospina is situated slightly posterior from and between the laterally exposed furcae. A distinct and isolated spinasternum is absent. Directly posterior to the mesospinal pit **spp2**, the sterna of the meso- and metathorax are flexibly connected by a lathy median sclerite **ms** (*Mediansklerit* in [13]), Fig. 1e). The slender and feather-shaped mesothoracic trochantin **ti2** articulates anteroventrally with the coxa **cx2**.

### Metathorax

In general, the morphology of the tergum and pleuron of the pterothoracic segments is similar. Compared to the mesopleuron, the anterior margin of the metepisternum **amest3** has a broader basis (Fig. 2c, e). Main differences in the morphology of the pterothoracic segments are related to the sterna. The sternum of the metathorax **st3** is trapezoid in shape. It is narrower but longer than the mesosternum (Fig. 1e). The posteromedian located furcal pit **fup3** is more or less U-shaped. Internally, the metafurcae **fu3** of each body side are joined in a short common stem **fs** (Fig. 2a, d). The laterally projecting metafurcal arms bear a lateral process **lfup**, a posterolateral process **plfup**, and an anterior process **afup** (Fig. 2c, e). A spina is absent in the metathorax.

### Thoracic musculature of *T. neglectus* and its homologization with that of other Neoptera

The thoracic muscles of *T. neglectus* are illustrated in Figs. 3 and 4. The detailed description of these muscles is provided in Table 1 containing origin, insertion and specific characteristics. In addition, Table 1 provides a hypothesis for the homology of the muscles of *T. neglectus* with the muscles generally reported from neopteran insects according to the nomenclature of Friedrich & Beutel [36]. In general, a thoracic muscle is treated as an individual unit when both origin and insertion and, in addition, the function of this specific muscle are different from other thoracic muscles found in the thorax. Muscles that possess several bundles are characterized through differently originating muscle parts running together in one tendon at a common insertion point (e.g. m16). On the other hand, muscles can run parallel but their origin and insertion is clearly separated nontheless having the same function. These muscles are treated as derivatives of a single muscle (e.g. m44, m45).

**Table 1:** List of thoracic muscles of the cave cricket *Troglophilus neglectus*, specifying origin and insertion of each muscle including noteworthy characteristics and corresponding figure in the article. Furthermore, homologization (Hom*) according to the nomenclature after [36] is provided

Present study	Hom*	Origin	Insertion	Remarks	Figure
Prothorax					
*dorsal longitudinal muscles*					
m1	Idlm1	median region of prophragma	dorsal area of occipital rim (close to m2)		3B
m2	Idlm3	prophragma (between m1 and m3)	cervical membrane		3B
m3	Idlm5	anterior dorsomedial pronotal region	lateral region of prophragma	flattened, broad	3C
*dorsoventral muscles*					
m4	Idvm1	anterior process of lateral cervical sclerite	dorsolateral area of occipital rim (ventrad of m5)	short, thin	3B
m5	Idvm2+3	posterior on inner face of lateral cervical sclerite	dorsolateral area of occipital rim	long, slim	3C
m6	Idvm5	anterior part of pronotum (near m8)	posterior part of lateral cervical sclerites near cervicopleural articulation point	fan-shaped, long thin tendon	3D
m7	Idvm10	laterodorsal face of profural arm	ventrolateral area of prophragma		3C
m8	Idvm13	dorsolateral area of pronotum (above cryptopleura)	trochantin	long thin tendon	3C
m9	Idvm16?	lateral region of pronotum (posterior to cryptopleura)	posterolateral procoxal rim (close to m26)	strongly developed	3D
m10	Idvm18	posterolateral region of pronotum	posterolateral procoxal rim (close to pleurocoxal joint)		3F
m11	Idvm19	lateral area of pronotum (posterior to cryptopleura, beneath m9)	trochanter (with m16)	strongly developed	3E
*sternopleural muscles*					
m12	Ispm5?	distal on ventral surface of profurcal arm	ventral part of anterior margin of mesepisternum	slender	3F
*pleurocoxal muscles*					
m13	Ipcm2	anterior procoxal rim	posterior face of anterior process of lateral cervical sclerite of opposite site (near cervicooccipital articulation point)	slender	3C
m14	Ipcm4	anterior margin of cryptopleura	anterior procoxal rim (close to m15)		3E
m15	Ipcm5	anterodorsal area of cryptopleura	anterior procoxal rim (close to pleurocoxal joint)		3E
m16	Ipcm8	anterolateral and anterodorsal area of cryptopleura	trochanter (with m11)	largest muscle in prothorax, strongly developed, 2 bundles	3D
*ventral longitudinal muscles*					
m17	Ivlm3	dorsal surface of profurcal arm	ventral area of occipital rim	strongly developed	3B
m18	Ivlm4	posterior margin of profurcal arm	anterolateral process of prospina		3B
m19	Ivlm6	posterior margin of profurcal arm (beneath m18)	anterior face of dorsolateral process of mesospina		3C
m20	Ivlm7	proximal at posterior margin of profurcal arm	anterior margin of mesofurcal arm		3E
m21	Ivlm8	posterior margin of posterolateral process of prospina	dorsal face of mesospina		3B
m22	Ivlm9	posterolateral process of prospina	anterior margin of mesofurcal arm (proximad of m20 & m37)		3D
*sternocoxal muscles*					
m23	Iscm1–1	lateral face of profurcal stem	anteromediad procoxal rim (mediad of m24)		3E, 4A, 4B
m24	Iscm1–2	anterolateral face of profurcal stem	anterior procoxal rim (close to trochantinocoxal articulation point)		3D, 4A, 4B
m25	Iscm1–3	medial face of profurcal stem and adjacent prosternum	anterior procoxal rim (laterad of m24)		3C, 4A, 4B
m26	Iscm2	ventral face of profurcal arm	posterolateral procoxal rim		4A, 4B
m27	Iscm3	distal on ventral face of profurcal arm	posterior procoxal rim on inner median process	slender	3E, 4B
m28	Iscm5	tip of anterolateral prospinal process	posterior procoxal rim on inner lateral process		3F, 4A, 4B
m29	Iscm7	lateral processi of prospina	anterior mesocoxal rim		3F, 4A, 4B
Mesothorax					
*dorsal longitudinal muscles*					
m30	IIdlm1	median region of prophragma	median region of mesophragma	several indistinct bundles as thin muscle layer	3B
*dorsoventral muscles*					
m31	IIdvm4+5	central region of mesonotum	posterior mesocoxal rim	two independent muscles sharing one insertion point	3E
m32	IIdvm6	dorsal edge of mesepimeron (ventrad of m31)	posterior mesocoxal rim (close to pleurocoxal joint)		3F
m33	IIdvm7	anterior region of mesonotum	trochanter (with m41 & m49)	largest muscle in mesothorax	3C
*tergopleural muscles*					
m34	IItpm10	epimeral face of mesopleural ridge	lateral region of mesonotum (ventrad of m32)	flattened	3F
*sternopleural muscles*					
m35	IIspm2	dorsal surface of mesofurca	ventral surface of mesopleural arm	poorly developed	3E
m36	IIspm6	posterior mesofurcal process	anterodorsal margin of metepisternum		3D
m37	IIspm?	anterior margin of mesofurcal arm (close to m20)	epimeral face of propleural ridge on cryptopleura	long thin tendon	3D
*pleurocoxal muscles*					
m38	IIpcm1	anterior margin of mesepisternum (close to m39)	trochantin		3F
m39	IIpcm2	inner anterodorsal part of anterior margin of mesepisternum	anterior mesocoxal rim		3F
m40	IIpcm3+4	episternal face of mesopleural ridge, few fibers from mesopleural arm	anterolateral mesocoxal rim	long, slender	3F
m41	IIpcm5	episternal face of mesopleural ridge and mesopleural arm	trochanter (with m33 & m49)		3E
*ventral longitudinal muscles*					
m42	IIvlm3	posterolateral process of mesofurcal arm	tip of anterior metafurcal process		3E
m43	IIvlm5	lateral face of posterior mesospinal process	medial face of anterior metafurcal process		3B
*sternocoxal muscles*					
m44	IIscm1–1	lateral at mesofurcal stem	anterior mesocoxal rim (close to trochantinocoxal articulation point)		3F, 4A
m45	IIscm1–2	anterior to mesofurcal stem at mesosternum	anterior mesocoxal rim (close to m44)		3E, 4A, 4B
m46	IIscm3	ventral face of mesofurcal arm	mesal mesocoxal rim		4B
m47	IIscm4	ventral face of mesofurcal arm (posterior to m46 & m49)	lateral mesocoxal rim (close to pleurocoxal joint)		3F, 4A, 4B
m48	IIscm5	ventrolateral and dorsolateral process of mesospina	posterior mesocoxal rim		3F, 4A, 4B
m49	IIscm6	ventral face of mesofurcal arm (anterior to m46 & m47)	trochanter (with m33 & m41)		4B
m50	IIscm7	posterior face of lateral processi of mesospina	anterior metacoxal rim		3F, 4A, 4B
Metathorax					
*dorsal longitudinal muscles*					
m51	IIIdlm1	median region of mesophragma	median region of metaphragma	several indistinct bundles as thin musle layer	3B
*dorsoventral muscles*					
m52	IIIdvm2	mesophragme and anterior part of metanotum	trochantin	runs partly behind m56	3C
m53	IIIdvm4	anterolateral region of metanotum	posterior metacoxal rim		3B
m54	IIIdvm5	anterolateral region of metanotum (dorsad of m53)	posterolateral metacoxal rim (close to m65)		3B
m55	IIIdvm6	osterolateral metacoxal rim (close to pleurocoxal joint)	dorsal epimeral face of metapleura (close to m57)		3C
m56	IIIdvm7	anterolateral region of metanotum (anterior to m54)	trochanter (with m63 & m68)	largest muscle in metathorax	3B
*tergopleural muscles*					
m57	IIItpm10	epimeral face of metapleura (dorsad of m55)	lateral region of metanotum	flattened	3C
*sternopleural muscles*					
m58	IIIspm2	dorsal surface of lateral metafurcal process	ventral surface of metapleural arm	strongly developed	3D
m59	IIIspm5	posterior face of metafurcal stem	intersegmental membrane between metathorax and abdominal pleura		3B
*pleurocoxal muscles*					
m60	IIIpcm1	anterior margin of metepisternum	trochantin		3D
m61	IIIpcm2	inner anterodorsal part of anterior margin of metepisternum (lateral to m60)	anterior metacoxal rim		3D
m62	IIIpcm3+4	dorsal metepisternum and dorsal episternal face of metapleural ridge, few fibers from metapleural arm	anterior metacoxal rim	well developed	3D
m63	IIIpcm5	dorsal part of metepisternum (dorsad of m62)	trochanter (with m56 & m68)		3C
*sternocoxal muscles*					
m64	IIIscm1	along lateral margin of metasternum	anterior metacoxal rim (close to trochantinocoxal joint)	broad origin	3D, 4A, 4B
m65	IIIscm2	posteroventral face of metafurcal stem	along inner posterior metacoxal rim	strongly developed, broad insertion	3C, 4A, 4B
m66	IIIscm3	ventral face of anterior and lateral metafurcal process	inner mesal metacoxal rim		3E, 4A
m67	IIIscm4	tip of posterolateral metafurcal process	lateral mesocoxal rim (close to pleurocoxal joint)	very thin and short	3C, 4A, 4B
m68	IIIscm6	distal at lateral metafurcal process	trochanter (with m56 & m63)		3F, 4B

The nomenclature of neopteran thoracic muscles presented by Friedrich & Beutel [36] provides a solid basis for homologizing thoracic muscles across insect groups. In some cases, however, the homologization of the thoracic muscles of *Troglophilus* with the muscles of the “generalized neopteran thorax“[36] proves to be difficult, because muscles are solely defined by their origin and insertion points. While we were able to largely homologize the thoracic muscles unambiguously, we will discuss some problematic cases in the following:

The **M. pronoto-trochantinalis anterior** (Idvm13) and **M. pronoto-trochantinalis posterior** (Idvm14) both share the same insertion point on the trochantin and have only a slightly different origins on the pronotum: Idvm13 originates from the anterior region of the pronotum, whereas Idvm14 arises from the central region of the pronotum [36]. In *Troglophilus*, the muscle m8 originates at the dorsolateral area of the pronotum slightly above the cryptopleura, inserting at the trochantin via a long and thin tendon. As m8 is the only muscle originating from the dorsal area of the pronotum it is questionable whether m8 is homologous to Idvm13 or Idvm14. Therefore, further criteria for homologization are necessary. A similar muscle with a long thin tendon is also present in other ensiferans [13]. According to Ander [13], the point of origin of this pronotal muscle has shifted from an anterior laterodorsal area above the cryptopleura to the lateral or central area of the pronotum behind the cryptopleura. Thus, the muscle m8 of *Troglophilus* is most likely homologous to Idvm13 according to the nomenclature of Friedrich & Beutel [36].

The **M. profurca-phragmalis** (Idvm10) is a common feature among major polyneopteran taxa [36, 48]. This muscle usually connects the profurca with the prophragma. However, in some orthopteran species, like in the grasshopper *Dissosteira carolina* (muscle 59) [56] or the stick grasshopper *Cephalocoema albrechti* (muscle 59) [57], Idvm10 has an insertion point shifted to the anterior part of the mesopleura. In *Troglophilus*, both conditions are present at the same time (m7 and m12). The muscle m7 is undoubtedly homologous to Idvm10 as it arises on the dorsal face of the profurca and inserts at the ventrolateral part of the prophragma. The second muscle (m12) takes a more horizontal course and arises from the ventral surface of the profurca inserting ventrally at the anterior margin of the mesepisternum. Because of their diverging courses and their differing origins on the profurca, the muscles m7 and m12 are most likely two separate muscles and not portions of a single muscle. Therefore, we conclude that muscle m12 of *Troglophilus* is homologous to **M. profurca-intersegmentalis posterior** (Ispm5) [36]. This assumption is also supported by the presence of serially homologues of m12 in the meso- and metathorax of *Troglophilus* (m36 and m59). Furthermore, a simultaneous presence of Idvm10 and Ispm5 is only known from Phasmatodea (*Megacrania tsudai*, *Carausius morosus*) and Embioptera (*Oligotoma saundersii*) [36]. In contrast to the morphology of *Troglophilus*, the muscle Ispm5 is attached to the peritreme in *Megacrania* [53] and *Oligotoma* [44], but to the intersegmental fold in *Carausius* [52]. These different attachment points cause uncertainties in regard to the homology of the muscle m12. Therefore, a question mark is added here (see Table 1).

In the generalized neopteran thorax, three pterothoracic dorsoventral muscles are attached to the posterior coxal rim [36]: **M. noto-coxalis anterior** (II/III dvm4), **M. noto-coxalis posterior** (II/IIIdvm5) and **M. coxa-subalaris** (II/IIIdvm6). In winged Neoptera, the muscles II/IIIdvm4 and II/IIIdvm5 originate at the central region of the nota, while II/IIIdvm6 inserts at the subalare. According to literature data [48, 49], the insertion point of II/IIIdvm6 is translocated to the lateral region of the nota in wingless Neoptera. This interpretation is consistent with the assumed tergal origin of the subalare, as proposed before [44, 58, 59]. In winged orthopterans, all three dorsoventral muscles are also well developed with the muscle II/IIIdvm6 inserting at the subalare. In contrast, the same muscle inserts at the epimeral face of the pleura in wingless Orthoptera: in the cave crickets *Troglophilus neglectus* (m32 and m55; present study) and *Diestrammena asynamora* (cx-em^2^) [46], in the New Zealand tree weta *Hemideina femorata* (Ab4) [60], in the apterous proscopiids *Cephalocoema albrechti* (90a and 120) [57], in morabine grasshoppers (99 and 129) [61], in wingless females of Pamphagidae, *Lamarckiana* sp*.* (depressor extensor muscle) [62], and also in micropterous species of Acrididae, e.g. *Barytettix psolus* (99 and 129) [63]. These findings are more consistent with the assumption of a pleural origin of the subalar sclerite, as suggested by other authors [40, 51, 64–66]. It is noteworthy that the hypothesis of a pleural origin of the basalar and subalar plates is exclusively based on developmental studies on orthopterans. With reference to Snodgrass [51], the aforementioned plates of nymphal Ensifera (*Gryllus*) and Caelifera (*Melanoplus*) are not yet differentiated from the pleura, and the M. coxa-subalaris (3E’ and 3E”) arises from the upper edge of the pterothoracic epimeron. Voss [41–43] who compared the thoracic musculature of different developmental stages of the house cricket *Acheta domesticus* also observed the epimeral insertion of the M. coxa-subalaris in the first instar (II and IIIpm6 in [41]; II and IIIldmv2 in [42, 43]), in which the basalar and subalar plates (*Pleuralgelenkplatten*) are not yet present.

Muscle m37 of *T. neglectus* is not described in Orthoptera or other insect taxa [59]. Due to its sternal origin at the anterior face of the mesofurca and its pleural insertion at the posterior edge of the cryptopleura, this muscle should be assigned to the sternopleural muscles [36]. Compared with the generalized neopteran thorax, muscle m37 is likely homologous to **M. mesofurca-intersegmentalis anterior** (IIspm7) with an insertion point shifted from the intersegmental membrane/ intersegmental sclerite to the posterior edge of the propleura. A muscle connecting the intersegmental sclerite between the pro- and the mesothorax with the mesothoracic furca is present in *Corydalus* (Megaloptera) [59]. In Mantodea, a muscle that arises on the prosternum near the prothoracic spina inserting at the metafurca, is apparently homologous to muscle IIspm7 [36, 59]. The specific traits of m37 in *Troglophilus* cannot be compared with the conditions reported from the aforementioned insect taxa. For this reason, we cannot homologize this muscle with any muscle listed by Friedrich & Beutel (see Table 1).

### Phylogenetically informative characters

**Fig. 5:**
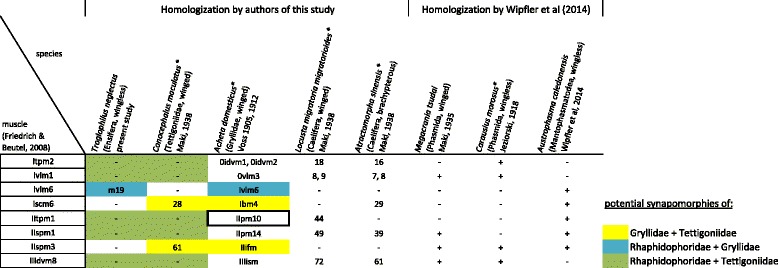
Phylogenetically informative muscle characters of ensiferans as compared with selected members of Caelifera and other wingless/winged representatives of Polyneoptera. Common characters (= potential synapomorphic traits) are indicated by color. Direct flight muscles, as indicated by Voss [41, 43], are framed by a rectangle. Species marked with an asterisk (*) bear different names in the respective cited publication (modified after [54] and [55])

The thoracic muscles found in *Troglophilus* are compared to that of a cricket, *Acheta domesticus* [40–43], and a bush-cricket, *Conocephalus maculatus* [44], in order to find similarities and differences between the major ensiferan groups represented by these species. Two fully winged locusts, the African Migratory Locust *Locusta migratoria migratorioides* [44] and European Migratory Locust *Locusta migratoria migratoria* [67], and a brachypterous representative, *Atractomorpha sinensis* [44], of the Caelifera, the sister group of Ensifera [68, 69], are also considered for comparison to delineate apomorphic and plesiomorphic traits. Moreover, further taxa of Polyneoptera, either having fully developed wings or being apterous, are also studied to draw reliable conclusions about the importance and effect of winglessness on the thoracic muscular system. The phylogenetically informative characters, which have a different manifestation in the Caelifera, are compiled in Fig. 5. A table providing the complete data set of the thoracic muscles of the aforementioned representatives is available as an additional data file (Additional file 2).

## Discussion

### Characters unique for cave crickets

**Fig. 6:**
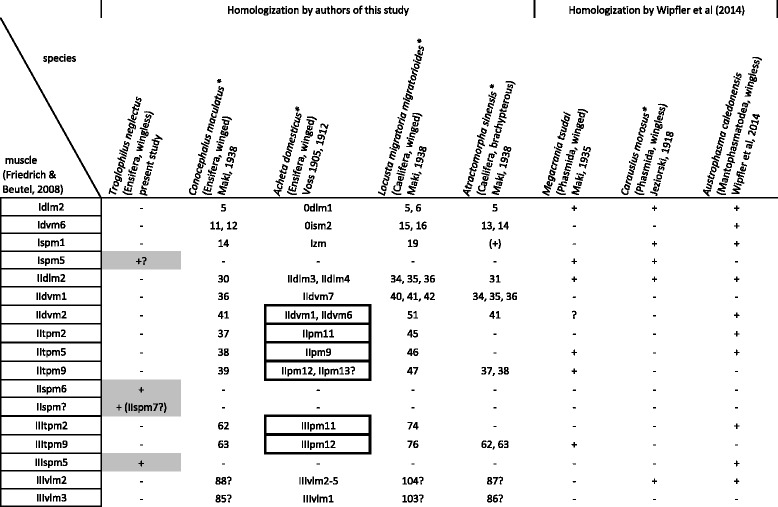
Unique muscular characters of Troglophilus neglectus as compared to other polyneopteran representatives. Potential positive apomorphies are indicated in light grey. Direct flight muscles, as indicated by Voss [41, 43], are framed by a rectangle. Species marked with an asterisk (*) bear different names in the respective cited publication (modified after [53] and [54])

Rhaphidophorids are generally considered as the morphologically most homogenous taxon within the Ensifera [13, 26]. Interestingly, rhaphidophorids are the only ensiferan subgroup for which no apomorphic character was reported in the cladistic analysis of Desutter-Grandcolas [21]. However, the thoracic muscular system of *T. neglectus* differs in significant points from that of other ensiferans, providing a number of potential autapomorphies (see Fig. 6). In general, the enlarged number of sternopleural muscles is a novelty for *Troglophilus*. In particular, the presence of m36 (IIspm6) and m37 (IIspm?) is unique within Orthoptera. *Troglophilus* is characterized by a largely reduced set of direct and indirect flight muscles. Both orthopteran representatives of the species-rich crickets (Gryllidae) and bush-crickets (Tettigoniidae) that we used for comparison are fully winged. In contrast, cave crickets completely lack wings. Thus, it is difficult to decide whether a flight muscle absent in *Troglophilus* is only a result of winglessness or represents an apomorphic character of Rhaphidophoridae. Since the ratio of flightless species to volant ones among orthopterans ranges between 30 and 60 % [1], the small taxon sampling of our study is insufficient to address this question.

It is particularly noteworthy that in *Troglophilus* the well developed musculature is important for operating the legs. These muscles are attached to the coxal rim or the trochanter and enable diverse movements of the legs. These muscles are either strongly developed, like Mm. noto-trochanteralis (m11, m33, m56), or their number is increased, like in the pro- and mesothoracic sternocoxal muscles scm1 (m23-25, m44-45). This strengthening of the sternocoxal muscles through multiplication is also reported from the wingless New Zealand tree weta *Hemideina thoracica* [60]. M. coxo-subalaris (II/IIIdvm6), which has an additional function as a flight muscle in winged insects [70], exclusively acts as leg retractor in *Troglophilus*. Additionally, *Troglophilus* has several sternopleural muscles that have not been described for other orthopterans. These include the serially homologous muscles m12 (Ispm5?), m36 (IIspm6) and m59 (IIIspm5) as well as the not homologized m37 (IIspm?). The connection of sternal and pleural elements by these muscles might lead to an enhanced movability of the thoracic segments (against each other), since there are no rigid connections of e.g. the pterothoracic sterna as in grasshoppers [13, 71]. Together with the strong leg musculature, the sternopleural musculature probably facilitates the scrambling movement of *Troglophilus* on cave walls and an increased jumping capability.

As suggested by authors of similar morphological studies [13, 72], the morphology of the thoracic sternum and associated sclerites in particular differs in decisive points between major ensiferan lineages. Including data on the thoracic skeletal anatomy of *Diestrammena asynamora* (Rhaphidophorinae) [45, 46] and *Macropathus filifer* (Macropathinae) [47] this specific character complex indeed provides some apomorphic traits for the Rhaphidophoridae. **Prothoracic spinasternum and prospina.** The characteristics of the prothoracic spinasternum and its internal protrusion, the prospina, have a unique appearance in rhaphidophorids. The prospinasternum of cave crickets is completely reduced externally (see Fig. 1e and [13]). Its presence is only noticeable by the existence of the prospina located in the membranous fold between the pro- and the mesosternum. In other ensiferan taxa, the prospinasternum is either exposed in the sternal intersegmental fold as a fully developed sclerite or merged with the posterior part of the prosternum or the anterior part of the mesosternum [13, 71, 72]. Also the star-shaped prospina, consisting of paired anterolateral and posterolateral processes and an unpaired anterior process, is a unique feature of rhaphidophorids. It has also been described in *Diestrammena asynamora* [45] and *Macropathus filifer* [47], two other representatives of cave crickets. In tettigoniids the prospina is triangular or t-shaped [72], when present. Voss [40] describes the prospina of *Acheta domesticus* as an irregular four-sided plate. The prospina of the mole cricket *Gryllotalpa vulgaris* is a long blade-like structure [73].

**Median sclerite between meso- and metasternum.** A narrow median sclerite, situated in a longitudinal arrangement between the sterna of the meso- and metathorax, is a typical feature of all rhaphidophorids [13]. This sclerite is frequently present in other ensiferan taxa, but the specific condition is different. In tettigoniids it can be rectangular or trapezoid, mostly spanning the whole width of the metasternum [72]. A triangular or semicircular sclerite is embedded at the anterior part of the metasternum in Anostostomatidae [13, 60], whereas in schizodactylids it is narrow and rectangular, inflexibly connecting meso- and metasternum ([71], unpublished observations for *Comicus* FL). Since the anatomical situation in rhaphidophorids is similar to that found in *Grylloblatta*, Ander [13] assumes that this sclerite is at least the posterior part of the mesothoracic spinasternum, since the mesospina is situated at the posterior end of the mesosternum right between the furcal apophyses. In contrast, Matsuda [59] and Naskrecki [72] refer to this sclerite as metathoracic presternum. As another alternative, Matsuda [59] characterizes the sclerite in question as the secondarily detached anterior part of the metathoracic basisternum. Due to these uncertainties, we simply refer to the sclerite as median sclerite **ms** following Ander [13].

**Metafurca.** The shape and specific structure of the metathoracic furca is another peculiarity of the thoracic skeleton of cave crickets. Rhaphidophorids possess a triramous furca with continuously tapered processes: an anterior, a lateral and a posterolateral one (see Fig. 2 and [45, 47]). Most other ensiferans have a biramous metafurca bearing a lateral and a posterior process [40, 72]. Like rhaphidophorids, the metafurca of Anostostomatidae has three processes, but the lateral one differs in shape from that of Rhaphidophoridae. In Anostostomatidae it is a flat, blade-like structure, termed apophysis wing, which directly projects beneath the pleural arm [60].

### Phylogenetic implications

**Fig. 7:**
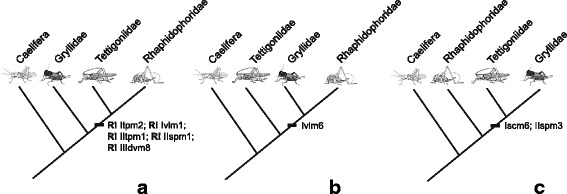
Informative characters of a comparative morphological study of the thoracic muscular system of representatives of Ensifera. The characters are mapped on the three competing hypotheses of the relationship between crickets (Gryllidae), bush-crickets (Tettigoniidae) and cave crickets (Rhaphidophoridae). Based on homologization in Table 1 (compiled in Additional file 2). R! indicates a reduced character in the respective taxa

The scarce information available for ensiferan thorax morphology is not yet sufficient for a cladistic analysis. However, the thoracic characters found in *Troglophilus neglectus*, *Acheta domesticus* (Gryllidae) and *Conocephalus maculatus* (Tettigoniidae) in comparison to other polyneopteran representatives (see Additional file 2) shows potential synapomorphies for certain subgroups within the Ensifera. As summarized in Fig. 7, the most parsimonious hypothesis of the phylogenetic position of cave crickets within the Ensifera supports a closer relationship to bush-crickets (Tettigoniidae) than to true crickets (Gryllidae). Hence, the hypothesis of ensiferan relationships favoured by the majority of authors (see Additional file 1) is also supported by thoracic muscle characters. Interestingly, all of the potential synapomorphies of Rhaphidophoridae and Tettigoniidae are negative character traits, i.e. reductions. This implies that the number of thoracic muscles decreases in a specific lineage among Ensifera, viz. Rhaphidophoridae + Tettigoniidae.

On the other hand, the alternative hypotheses also gain support by few characters of the thoracic musculature (Fig. 7). Gryllidae and Rhaphidophoridae share the presence of Ivlm6. However, this ventral longitudinal muscle frequently occurs within the Polyneoptera: in *Austrophasma caledonensis* (m26) [48], *Periplaneta americana* (101) [74], *Grylloblatta campodeiformis* (81) [75], *Oligotoma saundersii* (35) [44], and *Zorotypus hubbardi* (Ivlm6) [36]. Considering the thoracic muscular system, the presence of muscle Iscm6 and IIspm3 are the unique common characters of Gryllidae and Tettigoniidae. Nevertheless, Iscm6 is also present in the outgroup representatives *Atractomorpha sinensis* (29) [44] and *Austrophasma caledonensis* (m34) [48]. Muscle Iscm6 connects the profurca with the trochanter of the foreleg. In *Troglophilus*, the profurca is relatively short and does not extend beyond the opening of the coxa. This specific morphology would not allow lscm6 to reach the trochanter, which, from a functional point of view, could explain its secondary absence in *Troglophilus*. Although lacking in the representatives of the Caelifera, muscle IIspm3 appears to represent a common character of other polyneopteran taxa since it is present e.g. in Blattodea, *Periplaneta americana* (149) [74], Phasmatodea, *Carausius morosus* (IIildvm) [52] and *Megacrania tsudai* (148) [53], Mantophasmatodea, *Austrophasma caledonensis* (m51) [48], and Zoraptera, *Zorotypus hubbardi* (IIspm3) [36].

### The thorax of *Troglophilus neglectus* and the evolution of secondary winglessness in general

The consequence of wing reduction and flight loss largely affects thorax morphology in insects, both cuticular structures and the muscular system, which includes secondarily undifferentiated terga, less extensive phragmata and reduced or poorly developed dorsal longitudinal muscles (II/IIIdlm1, II/IIIdlm2), as well as the absence of wing base sclerites and associated wing-steering muscles [36, 60]. These distinctive traits are also found in the thorax of *Troglophilus*. In contrast to other wingless taxa like *Grylloblatta* [75] and the wingless morph of *Zorotypus* [36], the pleural arms in the pterothorax of *Troglophilus* are still well pronounced. Additionally, well developed pleural arms seem to be a common feature of Orthoptera, regardless the wing status, either fully winged [40, 56], micropterous [63] or wingless [46, 57]. In Mantophasmatodea, the well-developed pleural arms are explained by the climbing lifestyle among shrubs [48].

M. pleura-sternalis (II/IIIspm1), which is attached dorsally on the basalare and ventrally on the lateral part of the sternum, is thought to act as an extensor and flexor of the wing, and therefore is considered to be a direct flight muscle [56]. With the exception of Grylloblattodea and Mantophasmatodea, the general trend among wingless insects is the reduction of this muscle [48]. This trend is also observed within Orthoptera. In Caelifera, M. pleura-sternalis is present in the meso- and metathorax of winged locusts [44, 56], whereas it is absent in the micropterous Mexican grasshopper *Barytettix psolus* [63], and also reduced in wingless Proscopiidae [57] and morabine grasshoppers [61]. The assumption that M. pleura-sternalis is at least present in the mesothorax of Ensifera is based on the description of a single cricket species [41–43]. After investigation of several additional ensiferan species, we can now reliably conclude that muscle IIspm1 is only present in Grylloidea, e.g. *Acheta domesticus* (IIpm14) [41] and *Gryllus campestris* (ls-es^1^) [46], and in the mole cricket *Gryllotalpa gryllotalpa* (LS-EP_2_) [76]. The muscle is lacking in the meso- and the metathorax of the cave cricket *Troglophilus*, the schizodactylid *Comicus calcaris* (unpublished observations FL) and the winged bush-cricket *Conocephalus maculatus* [44]. This reduction of muscle spm1 in the pterothorax, especially in Tettigoniidae, might be a phylogenetically informative character, which needs to be tested in a future cladistic analysis based on an enlarged taxon sampling.

In the pterothorax of *Troglophilus*, dorsal longitudinal (II/IIIdlm2), dorsoventral (II/IIIdvm1) and tergopleural muscles (tpm) are absent, muscles that are indirectly or directly involved in flying [36, 48]. Most notably, the number of wing-steering tergopleural muscles is reduced, as has also been reported from other wingless taxa, e.g. Phasmatodea [49, 52] or Orthoptera [57, 60]. The only tergopleural muscle retained in both pterothoracic segments of *Troglophilus* is M. epimero-subalaris (II/IIItpm10). In winged species, this muscle connects the dorsal part of the epimeron with the subalar sclerite [36]. As in *Troglophilus*, the insertion point of tpm10 is translocated to the notum in wingless species of Phasmatodea [49] or Mantophasmatodea [48].

Regarding the two major lineages of Orthoptera, Caelifera (grasshoppers) and Ensifera (katydids and crickets), muscle tpm10 is only known to exist in the meso- and metathorax of ensiferan taxa [41, 44, 76]. Only Maki [44] described a muscle tpm10 in the mesothorax of the African Migratory Locust *Locusta migratoria migratorioides* (see Additional file 2), but neither Albrecht [67] observed this muscle in the European Migratory Locust *Locusta migratoria migratoria*, nor did Snodgrass [56] in his study about the thoracic morphology of the Carolina Grasshopper *Dissosteira carolina*. In general, the number of tergopleural muscles that have been described for *Locusta* (II/IIItpm1, II/IIItpm2, II/IIItpm5, II/IIItpm9 and IItpm10) is exceptionally large [44]. Somewhat surprisingly, only M. epimero-axillaris tertius (II/IIItpm9) is known in *Locusta migratoria migratoria* (85 and 114) [67], *Dissosteira carolina* (85 and 114) [56], the wingless morabine grasshoppers (tergopleural muscle) [61], and even in the brachypterous *Atractomorpha sinensis* (37/38 and 62/63) [44]. In wingless Caelifera, like *Lentula callani* [77] and *Cephalocoema albrechti* [57], even this muscle is reduced and not a single tergopleural muscle has ever been reported. In summary, the distinctive set of tergopleural muscles differs significantly between Caelifera and Ensifera and the role of these muscles after wing loss is markedly dissimilar.

In Euphasmatodea (the majority of extant stick insects) on the other hand, thoracic morphology of wingless species largely resembles conditions found in Ensifera. Klug [49] observed a significantly reduced set of tergopleural muscles in wingless stick insects, only consisting of muscles II/IIItpm10 and II/IIItpm13 (tpm13 is a unique muscle of Phasmatodea). These partly comparable patterns imply that the mechanism and morphology of secondary winglessness may follow similar routes in closely related taxa. In contrast, in Embioptera (webspinners), the assumed sister taxon of Phasmatodea [69], the set of tergopleural muscles (II/IIItpm1, II/IIItpm5, II/IIItpm6, II/IIItpm7, II/IIItpm10; homologized in [48]) does not differ between winged males and wingless females of the same species [78, 79].

Another pattern providing support for the assumption of similar evolutionary trajectories in closely related taxa can be observed in the entirely wingless Xenonomia [80] comprising heelwalkers (Mantophasmatodea) and ice crawlers (Grylloblattodea). Here, the set of tergopleural muscles is different from that of wingless representatives of Orthoptera, Phasmatodea or Embioptera. *Grylloblatta campodeiformis* (Grylloblattodea) is characterized by a set of IItpm1/5 and IIItpm1/5 [75] (homologized in [36]). Based on the description of Klug [49], *Austrophasma caledonensis* (Mantophasmatodea) exhibits the same set of tergopleural muscles in the pterothorax, IItpm1/5 and IIItpm1/5. According to the reinvestigation of the same species [48] a considerably higher number of tergopleural muscles is reported: IItpm1/2/3/4/5/?10 and IIItpm1/2/3/4/5/?10. These studies used different µCT data sets for analysis. Depending on the quality of the data sets, it is possible that some muscles were initially overlooked, e.g. tpm10 characterized as a flat muscle closely fitting the skeletal elements. Nevertheless, muscle tpm1 in Klug [49] and the four muscles tpm1/2/3/4 described for *Austrophasma* by Wipfler et al. [48] are located in the same small area between the anterior part of the tergum and the dorsal part of the pleural ridge. A further explanation of these striking differences might lie in the different life stages or sexes investigated in both studies. Klug [49] examined a nymphal stage of unknown sex of *Austrophasma caledonensis*, whereas in the study of Wipfler et al. [48] no explicit information about the developmental stage or the sex of the investigated specimens is provided. However, studies about the postembryonic development of the flight musculature of hemimetabolous insects show that these muscles are less developed in early nymphal stages, significantly increasing in size during their ontogenesis [81–84]. Other studies comparing the thoracic musculature report a differing number of muscles in nymphs and adults of the same species [41, 42, 85]. In consequence, the presence of tpm1 and tpm5 in the meso- and metathorax of Grylloblattodea and Mantophasmatodea might still be considered a synapomorphic character of both taxa.

Principally, the flight ability and performance of insects also depend on the total mass of flight muscles present, and not only on the concrete set of direct and indirect flight muscles [84]. Nonetheless, the concrete set of tergopleural muscles differs between major insect groups [36]. Regarding the Orthoptera, their flight ability and performance become of secondary importance, since many species primarily move by jumping. In these cases, wings are mainly used to control the direction and trajectory during the jumping process [5, 86]. For instance, the house cricket *Acheta domesticus* [41], with a set of IItpm1/2/5/9/10 and IIItpm1/2/5/9/10, and the tettigoniid *Conocephalus (Anisoptera) maculatus* [44], with a reduced set of IItpm2/5/9 and IIItpm2/9/10, exhibit similar flight capability [44, 86]. On the other hand, the absence of specific tergopleural muscles as in the brachypterous gaudy grasshopper *Atractomorpha sinensis* [44] having only a single duplicated tergopleural muscle in the meso- and metathorax (II/IIItpm9) causes a low vagility [87]. In contrast, *Sipyloidea sipylus*, a winged stick insect, only has the ability to control its speed and trajectory during free fall with a set of six different metathoracic tergopleural muscles in the flight apparatus (tpm1/3/4/6/9/10) [49, 88]. In conclusion, there appears to be no correlation between an increased number of pterothoracic tergopleural muscles and an enhanced flight capability. However, an extremely reduced set of tergopleural muscles does consequently lead to the inability to fly.

Anatomical structures that are no longer used will be reduced in the course of evolution, and the degree of reduction can be an indicator of the time elapsed [89]. Nevertheless, conservative anatomical elements can be retained although associated traits of the periphery are lost [90]. As we have outlined, the loss of wings in insect groups like Orthoptera, Xenonomia [48] or Phasmatodea [49] has been followed by a number of anatomical adaptations of skeletal and muscular elements in the thorax. The insect lineages compared above exhibit significantly different evolutionary histories in regard of the time span since wing loss, affecting the degree of reduction or anatomical adaptations towards flightlessness. The radiation of Rhaphidophoridae began at least 140 million years ago [16, 19]. Thus, the Rhaphidophoridae may represent the oldest exclusively wingless lineage within Ensifera [19], and wing loss occurred most probably in the last common ancestor (autapomorphy) of all Rhaphidophoridae. The likewise wingless Xenonomia, heelwalkers (Mantophasmatodea) and ice crawlers (Grylloblattodea), are roughly the same age as the Rhaphidophoridae [69]. We have demonstrated that the thoracic musculature differs significantly in both lineages. In comparison, the wingless representatives of Euphasmatodea are significantly younger. The diversification of their major extant lineages took place during a period of about 20 million years, and presumably started after the Cretaceous-Tertiary boundary ~66 million years ago [91, 92]. The thoracic musculature of wingless Ensifera, Rhaphidophoridae in particular, is most similar to the conditions found in the much younger wingless representatives of Euphasmatodea than in the equally old Xenonomia, refuting any dependency between level of reduction and evolutionary time. This might be explained by the degree of correlation of the structures in question to other, still adaptive features [89].

## Conclusions

Secondary winglessness, a widespread phenomenon among pterygote insects, largely affects the thoracic anatomy including skeletal structures and the muscular system. By comparing the thoracic morphology of various wingless representatives of Polyneoptera, we demonstrate that anatomical adaptations towards flightlessness, especially regarding the flight musculature, are highly homogenous within major lineages, viz. Ensifera, Caelifera, Xenonomia, or Euphasmatodea. However, in most cases these specific adaptations are strikingly different between the aforementioned taxa indicating a markedly dissimilar role of these muscles after wing loss.

The thoracic morphology of Ensifera is a highly structured character complex whose investigation is a worthwhile endeavor, leading to a deeper understanding of functional adaptations during the evolution of Ensifera in general. We have shown that the thoracic morphology can be a valuable source for characterizing individual ensiferan taxa, providing a number of potential apomorphies for cave crickets (Rhaphidophoridae). Based on our comparison with other ensiferans, we can provide arguments for a closer relationship of Rhaphidophoridae to Tettigoniidae, rather than to Gryllidae. These findings are consistent with previous assumptions [19, 21, 22].

## Additional files

Additional file 1:
**Competing hypotheses of the relationships between true crickets (Gryllidae), bush-crickets (Tettigoniidae) and cave crickets (Rhaphidophoridae) following different authors.** Further ensiferan taxa are excluded in this scheme. Studies marked by an asterisk (*) are based on formally cladistic analyses, studies tagged with a triangle include fossils. (TIF 176 kb) Additional file 2:
**Thoracic muscles of different representatives of Polyneoptera homologized following nomenclature by [** 36 **].** (XLSX 21 kb)
