# Supplementary figures and images for: The thorax of the cave cricket Troglophilus neglectus: anatomical adaptations in an ancient wingless insect lineage (Orthoptera: Rhaphidophoridae)
Source: BMC Evol Biol. 2016 Feb 18;16:39. doi: 10.1186/s12862-016-0612-5 (PMC4758143; doi:10.1186/s12862-016-0612-5)

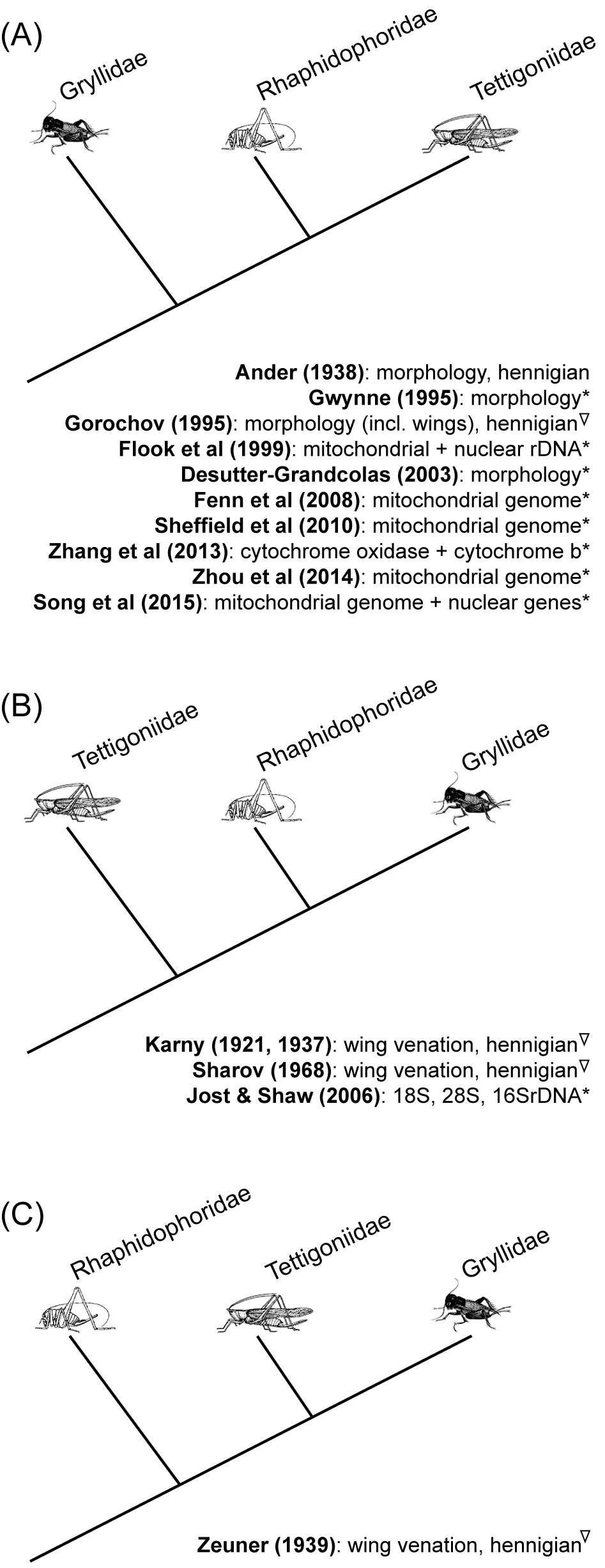

Supplement: Additional file 1: — Competing hypotheses of the relationships between true crickets (Gryllidae), bush-crickets (Tettigoniidae) and cave crickets (Rhaphidophoridae) following different authors. Further ensiferan taxa are excluded in this scheme. Studies marked by an asterisk (*) are based on formally cladistic analyses, studies tagged with a triangle include fossils. (TIF 176 kb) [file 12862_2016_612_MOESM1_ESM.tif]
